# Fusarium Endophthalmitis, Unusual and Challenging Infection in an Acute Leukemia Patient

**DOI:** 10.1155/2018/9531484

**Published:** 2018-03-13

**Authors:** Mehmet Baysal, Elif Umit, İbrahim Bekir Boz, Onur Kırkızlar, Muzaffer Demir

**Affiliations:** ^1^Department of Hematology, Faculty of Medicine, Trakya University, Edirne, Turkey; ^2^Deparment of Internal Medicine, Faculty of Medicine, Trakya University, Edirne, Turkey

## Abstract

Invasive fungal infections bring serious mortality and morbidity during the treatment of acute myeloid leukemia. Especially, mold infections are challenging, and each case is unique in feature. These cases are usually fatal, and there is no consensus regarding optimal treatment. AML patients receive antifungal prophylaxis and may further require IFI (invasive fungal infection) treatments, but fusarium mold infections are often unrecognized and could be overlooked. In this case report, we try to emphasize the importance of this infection with a high-risk AML patient.

## 1. Introduction

Fusarium species are generally present in soil and organic debris, and they are common pathogens of plants [1]. Though they are occasionally responsible in allergic reactions in immunocompetent individuals and in immunocompromised individuals (especially in patients with hematological malignancies and transplant recipients due to prolonged and profound neutropenia after treatment with highly cytotoxic chemotherapy), they may be the cause of invasive fungal infections [2]. *Aspergillus* spp. is the most common pathogen of invasive fungal diseases followed by fusarium spp [3]. Fusarium infection may arise through penetrating skin injuries or aspiration of airborne pathogens and can cause superficial, locally invasive, and even disseminated infections in immunocompromised patients [4]. Here, we present a primary refractory acute myeloid leukemia patient presenting with retinal fusarium endophthalmitis.

## 2. Case Presentation

A 28-year-old male patient admitted to our hospital with fatigue starting over three weeks. He had no history of any chronic disease and was experiencing night sweats 2 or 3 times a week before. He did not report use of any medicine or herbal products. Physical examination was unremarkable. His laboratory workup showed hemoglobin level 9.9 g/dl, leucocyte 5560 mm^3^, and platelets 76.000 mm^3^, and LDH level was slightly elevated with 568 U/L (reference range 0–236 U/L). Other biochemical and hematological parameters were insignificant. Atypical mononuclear cells were observed on peripheral blood smear. Blood marrow aspiration and biopsy was performed, and blood marrow aspiration showed 38% blastic infiltration. Flow cytometry revealed a blastic population with CD13, CD33, CD117, and clear MPO positivity which led to the diagnosis of AML. Under prophylaxy with posaconazole, intensive treatment with 7 + 3 (cytarabine + idarubicin) for remission induction was followed with FLAG-IDA (fludarabine, cytarabine, and idarubicin) due to remission failure. Since this patient was undergoing intensive chemotherapy and had a high probability of developing IFIs, serum galactomannan level was routinely obtained and monitored twice a week. Serial galactomannan measurements were negative. On the 11th day of chemotherapy, patient presented with fever, regarded as febrile neutropenia. Blood and urine cultures were obtained, and empirical piperacillin tazobactam was started. Fever persisted and antibiotics were switched to meropenem; cultures were repeated, still negative; and prophylactic posaconazole was switched to empirical voriconazole due to the finding of a possible invasive fungal infection determined with thorax tomography. During these interventions, the patient's physical examination was totally unremarkable besides fever episodes. On the fourth week of second line treatment, remission was not obtained, a full-match sibling was determined as eligible for allogeneic transplantation and clofarabine-cytarabine was selected for bridging therapy under the coverage of voriconazole. Before the initiation of chemotherapy, the patient complained of blurred vision with the right eye. Fundoscopic exam showed signs of bilateral retinal hemorrhage. Local antibiotic and corticosteroid treatments were initiated. Cranial and orbital MRI showed leukemic infiltration of the thalamus and right eye. Conjunctival and intraorbital sample cultures showed fusarium infection on the right eye. Liposomal amphotericin B was added to voriconazole for 3 weeks, and a control orbital MR scan was performed showing progression of infection. Clinically, intractable severe edema of the eye with progressive infection could be controlled only by enucleation of the right eye. Pathological evaluation of the enucleated eye showed fusarium hyphae with hyaline and septate filaments which dichotomize in abrupt and right angles.

After the debridement of the infected tissue and dual antifungal treatment, the patient was stable in regard of the infection. He underwent allogeneic transplantation and died 21 days after the transplantation due to pneumosepsis. The course of the disease is depicted in Table 1.

## 3. Discussion

Clinical manifestations of fusarium are broad. Patients with fusarium can present with skin lesions, pneumonic infiltrates, and retinal involvement or dissemination. Prolonged neutropenia poses a risk for fungal infections in acute myeloid leukemia patients treated with chemotherapy and stem cell transplantation. Although the exact frequency of fusariosis is less than 1% in patients with hematologic malignancies, the frequency is trending [5]. Current strategies regarding leukemia patients undergoing chemotherapy include using primary prophylaxis with mold-active agents. Early initiation of antifungal agents reduced the fungal infection-related mortality rates and documented invasive fungal infections [6]. However, breakthrough, resistant, and rare fungal infections become troublesome for clinicians.

Fusarium species tends to invade vitrea and often causes ocular involvement. This invasion is accompanied by endopthalmitis. Fusarium infection can be transmitted through breakdown of skin, with the use of tap water for personal hygiene, or can be airborne. In our patient, there were no skin lesions prior to endopthalmitis, and we suspected the use of tap water for daily care. In disseminated diseases, usually skin lesions with reddish macules and papules with central necrosis can be seen. Skin lesions frequently occur on extremities. In patients with pulmonary involvement, infiltrates, cavitary lesions, or ground glass densities can be seen. Unfortunately, these lesions are nonspesific for fusarium infection. *Aspergillus* infections also cause similar lung lesions.

There are several case reports mentioning fusarium infection in acute leukemia patients. The outcome of these patients is often poor despite heavy treatments [3, 7, 8]. One possible sign of fusarium infection in these patients is the skin lesions preceding dissemination. In a recent study, febrile neutropenic patients with skin lesions have a higher risk of invasive fungal infection [9]. In another study, antimold prophylaxis with azoles (posaconazole and voriconazole) decreased the incidence of fusariosis in high-risk hematologic patients with superficial skin lesions.

Treatment of fusariosis is often complicated and challenging. Liposomal amphotericin B should be considered as first line of treatment. (Given 3–5 mg/kg intravenous qd) Voriconazole treatment may be an alternative (6 mg/kg IV bid followed by 4 mg/kg bid as maintenance dose) [10]. Combination therapies may be used in systemic fusariosis, as systemic administration of voriconazole and amphotericin B or local voriconazole and systemic amphotericin B [5]. However, clinical significance of combination regimens remains unclear due to publication bias and lack of clinical trials. Duration of treatment depends on the patient's response to therapy, underlying disease, and resolution of neutropenia. Using of antifungal drugs as secondary prophylaxis should be considered in patients with a history of fusariosis or other fungal infections. Besides medical interventions, vitrectomy and enucleation was considered and performed like the case we presented. Surgical approaches may be used to prevent dissemination but usually found to be ineffective. Invasive and opportunistic fungal infections play a major problem for treatment of myeloid leukemia patients. Although prophylactic antifungal treatment shows benefit in reducing the risk of fungal infections, atypical and fusarium-like species can be encountered in this group of patients.

## 4. Conclusion

Fusarium causes serious morbidity and mortality and may mimic aspergillosis. Targeted antibiotherapy should be the main goal of treatment, and isolation of responsible strains should be supported in every immunocompromised patient.

## Figures and Tables

**Table 1:** Follow-up and course of the disease.

Time	Diagnosis	+1 month	+3 months	+4 months	+5 months	+6 months
Event	AML	Refractory AML	Refractory AML, probable fungal infection with thorax CT	Refractory AML blurred vision with the right eye—infiltration in the thalamus and right eye	Refractory AML enucleation and debridement of the right eye	Allogeneic stem cell transplant from HLA-matched sibling

Treatment	Posaconazole 200 mg (5 mL) PO TID remission induction 7 + 3 (cytarabine + idarubicin)	Posaconazole 200 mg (5 mL) PO TID remission induction FLAG-IDA (fludarabine-cytarabine-idarubicin)	Voriconazole 6 mg/kg IV q12 hr for first 24 hours, then 4 mg/kg IV q12 hr bridging therapy for allogeneic stem cell transplantation (clofarabine-cytarabine)	Voriconazole 4 mg/kg IV q12 hr + liposomal amphotericin B 5 mg/kg IV qday	Voriconazole 4 mg/kg IV q12 hr + liposomal amphotericin B 5 mg/kg IV qday	Died + 21 day of transplantation due to pneumosepsis

## References

[1] Cooke N. S., Feighery C., Armstrong D. K. B., Walsh M., Dempsey S. (2009). Cutaneous fusarium solani infection in childhood acute lymphoblastic leukaemia. *Clinical and Experimental Dermatology*.

[2] Garnica M., Oliveira da Cunha M., Portugal R., Maiolino A., Colombo A. L., Nucci M. (2015). Risk factors for invasive fusariosis in patients with acute myeloid leukemia and in hematopoietic cell transplant recipients. *Clinical Infectious Diseases*.

[3] Delia M., Monno R., Giannelli G. (2016). Fusariosis in a patient with acute myeloid leukemia: a case report and review of the literature. *Mycopathologia*.

[4] Nucci M., Anaissie E. (2007). Fusarium infections in immunocompromised patients. *Clinical Microbiology Reviews*.

[5] Nucci M., Garnica M., Gloria A. B. (2013). Invasive fungal diseases in haematopoietic cell transplant recipients and in patients with acute myeloid leukaemia or myelodysplasia in Brazil. *Clinical Microbiology and Infection*.

[6] Ethier M. C., Science M., Beyene J., Briel M., Lehrnbecher T., Sung L. (2012). Mould-active compared with fluconazole prophylaxis to prevent invasive fungal diseases in cancer patients receiving chemotherapy or haematopoietic stem-cell transplantation: a systematic review and meta-analysis of randomised controlled trials. *British Journal of Cancer*.

[7] Jossi M., Ambrosioni J., Macedo-Vinas M., Garbino J. (2010). Invasive fusariosis with prolonged fungemia in a patient with acute lymphoblastic leukemia: case report and review of the literature. *International Journal of Infectious Diseases*.

[8] Yoshida M., Kiyota N., Maruyama K. (2017). Endogenous fusarium endophthalmitis during treatment for acute myeloid leukemia, successfully treated with 25-gauge vitrectomy and antifungal medications. *Mycopathologia*.

[9] Aiempanakit K., Naorungroj S., Chiratikarnwong K., Auepemkiate S., Apinantriyo B. (2017). Risk factors for invasive fungal infection among thai oncologic patients with febrile neutropenia and cutaneous presentation: a 5-year retrospective study in Southern Thailand. *Asian Pacific Journal of Cancer Prevention*.

[10] Al-Hatmi A. M. S., Bonifaz A., Ranque S., Sybren de Hoog G., Verweij P. E., Meis J. F. (2017). Current antifungal treatment of fusariosis. *International Journal of Antimicrobial Agents*.

